# *Lactiplantibacillus plantarum* ELF051 Alleviates Antibiotic-Associated Diarrhea by Regulating Intestinal Inflammation and Gut Microbiota

**DOI:** 10.1007/s12602-023-10150-x

**Published:** 2023-08-28

**Authors:** Wei Liang, Yansong Gao, Yujuan Zhao, Lei Gao, Zijian Zhao, Zhongmei He, Shengyu Li

**Affiliations:** 1 College of Chinese Medicinal Material, Jilin Agricultural University, Changchun, 130118 China; 2 Institute of Agro-Food Technology, Jilin Academy of Agricultural Sciences, No. 1363 Sheng-Tai Street, Changchun, 130033 China

**Keywords:** Antibiotic-associated diarrhea, Gut microbiota, Intestinal inflammation, *Lactiplantibacillus plantarum*, Short-chain fatty acids

## Abstract

Probiotics are widely recognized for their ability to prevent and therapy antibiotic-associated diarrhea (AAD). This study was designed to evaluate *Lactiplantibacillus plantarum* ELF051 ability to prevent colon inflammation and its effect on gut microbial composition in a mouse model of AAD. The mice were intragastrically administered triple antibiotics for 7 days and then subjected to *L. plantarum* ELF051 for 14 days. The administration of *L. plantarum* ELF051 ameliorated the pathological changes in the colon tissue, downregulated interleukin (IL)-1β and tumor necrosis factor (TNF)-α, and upregulated IL-10, and increased the intestinal short-chain fatty acids (SCFAs) level. *Lactiplantibacillus plantarum* ELF051 also regulated the Toll-like receptor/myeloid differentiation primary response 88/nuclear factor kappa light chain enhancer of activated B cells (TLR4/MyD88/NF-κB) and the phosphatidylinositol 3-kinase/protein kinase B/ NF-κB (PI3K/AKT/ NF-κB) inflammatory signaling pathways. 16S rRNA analyses showed that *L. plantarum* ELF051 increased the abundance and diversity of gut bacteria, restoring gut microbiota imbalance. A Spearman’s rank correlation analysis showed that lactobacilli are closely associated with inflammatory markers and SCFAs. This work demonstrated that *L. plantarum* ELF051 can attenuate antibiotic-induced intestinal inflammation in a mouse AAD model by suppressing the pro-inflammatory response and modulating the gut microbiota.

## Introduction

Antibiotic-associated diarrhea (AAD) is defined as otherwise unexplained diarrhea that occurs in association with the administration of antibiotics. Most known antibiotics could induce AAD, with ampicillin, cephalosporins, and clindamycin being the most serious offenders, leading to an incidence of AAD ranging from 5 to 30% when any of the above antibiotics are used [1, 2]. Antibiotic therapy may induce AAD by disrupting the gut microbiota, altering the intestinal short-chain fatty acids (SCFAs) content, and changing the intestinal structure and barrier function [3]. Untreated AAD may result in pseudomembranous enteritis which has 15–24% mortality [4]. Treatments for AAD include antibiotic withdrawal, targeted antibiotic therapy, and oral microbiotics. However, none of these is very effective, and some of them might even be harmful [5]. Therefore, a new and effective AAD treatment is urgently required.

Probiotics have been studied for their health promoting effects, including influencing the composition and function of the gut microbiota, modulating the immune response, exhibiting antimutagenic effect and anti-cancer properties, and lowering serum cholesterol [6]. In recent years, increasing evidence has shown that probiotics also have beneficial impact on alleviating symptoms of AAD. Numerous randomized, double-blind, controlled clinical trials have provided evidence that probiotics, including *Bifidobacterium lactis*, *B. animalis*, *Lactobacillus casei*, *L. plantarum*, and *L. rhamnosus*, exert positive effects on modulating the intestinal microbiota and preventing and treating AAD [7–9]. Animal studies have also demonstrated that consumption of a mixture of *Lactobacilli* species (JUP-Y4) not only promoted recovery from antibiotic-induced gut dysbiosis but also enhanced the function of the gut barrier, and lowered levels of circulating endotoxin in mice [10]. The specific molecular mechanisms underlying the treatment and prevention of AAD by probiotics primarily involve the following aspects: (i) Modulating the gut microbiome. As direct evidence, many results show that AAD in mice is related to the changes in normal intestinal microbiota, which is mainly manifested by the reduction of beneficial bacteria and the increase of potential pathogens. The administration of probiotics has been proved to regulate the disorder of gut microbiota [11]. (ii) Enhancing the intestinal immune response. Probiotics have been proved to enhance humoral immunity response by increasing the cells secreting IgG, IgM, and IgA [12, 13]. (iii) Improving intestinal barrier function. Probiotics can increase mucin secretion, upregulate ZO-1 and occludin protein synthesis, thus repairing intestinal permeability caused by AAD [14]. (iv) Maintaining normal levels of SCFAs. Antibiotic-mediated gut microbiome remodeling results in significant alterations to intestinal metabolomes. Probiotic supplementation can significantly increase SCFAs, contributing to the improvement of intestinal health [15].

AAD is often accompanied by systemic inflammation, characterized by an increase in pro-inflammatory cytokines and a decrease in anti-inflammatory cytokines, indicating that anti-inflammatory may be a potential mechanism for probiotics to alleviate AAD [16]. Previous studies have confirmed that gastric perfusion of *L. plantarum* 2–33 into AAD mice can significantly increase the levels of anti-inflammatory factors IL-4 and IL-10 and reduce the levels of pro-inflammatory factors TNF-α and IFN-γ. Similar results were observed with probiotics *B. animalis* subsp. *lactis* XLTG11 [15] and *Bacillus subtilis* DU-106 [17] in regulating cytokine levels in diarrhea mice. However, while existing research has primarily focused on cytokine levels, a deeper understanding of the mechanisms underlying intestinal inflammation in AAD is lacking. Therefore, this study is mainly to carry out the protective effect of probiotics on intestinal inflammation in the development of AAD.

*Lactiplantibacillus plantarum* ELF051 is a potential probiotic isolated from Kimchi. Our previous research has proved that *L. plantarum* ELF051 significantly inhibits the growth of *Clostridium difficile* (data not shown), indicating its potential therapeutic effect on AAD. However, the precise mechanism underlying of *L. plantarum* ELF051 exerts effects in AAD mice remains unclear. Therefore, the present study was conducted to investigate the potential mechanisms of *L. plantarum* ELF051 in alleviating the progression in a AAD mouse model, by reducing colonic pathological changes, restoring SCFAs levels, modulating inflammatory cytokines, regulating inflammatory signaling pathways, and correcting gut microbiota imbalance.

## Materials and Methods

### Bacterial Strains and Culture Methods

*Lactiplantibacillus plantarum* ELF051 was incubated in De Man, Rogosa, and Sharpe (MRS) medium at 37 °C for 17 h, centrifuged at 3000 × g for 15 min, washed thrice with phosphate-buffered saline (PBS; pH 7.4), and resuspended in saline and the viable count of the bacterial solution was adjusted to 1.0 × 10^9^ CFU/mL, and kept at 4 °C.

### Animal Experiment Design

Male C57BL/6 mice (20 ± 2 g, *n* = 30) were obtained from Liaoning Changsheng Biotechnology Co., Ltd. (Benxi, China). All mice were adaptively fed under standard conditions over 7 days. Mice were randomly divided into three groups (*n* = 10/group): control, model, and ELF051 groups. From day 1 to day 7, the control group received physiological saline via gavage. Meanwhile, the model and ELF051 groups were administered a mixed antibiotic solution consisting of 500 mg/kg amoxicillin (Henan Puxin Biotechnology Co., Ltd; Henan, China), 400 mg/kg clindamycin (Anhui Lianyi Pharmaceutical Co. Ltd; Fuyang, Anhui, China), and 350 mg/kg streptomycin (Chongqing Xinjiheng Pharmaceutical Co. Ltd; Chongqing, China) via gavage with a volume of 0.2 mL [17]. From day 8 to day 21, the ELF051 group received *L. plantarum* ELF051 at a dosage of 0.1 mL/10 g via gavage, while both the control and model groups received an equal volume of saline. All animal experiments complied with the institutional animal care regulations of Jilin Academy of Agricultural Sciences (approval number: SCXΚ2020-0001).

After the final treatment, the mice were fasted for 12 h, and then anesthetized with ether. Subsequently, blood, feces, cecum, and colon samples were collected. The mice’s feces, cecal contents, and a portion of the colon were promptly frozen in liquid nitrogen and stored at − 80 °C for subsequent analysis. The remaining colon samples were preserved in Cano’s fixative for further investigations.

### Histological Analysis

The fixed colons were dehydrated with ethanol, embedded in paraffin, cut into sections 4 μm thick, and stained with hematoxylin and eosin (H&E) [18]. Pathological changes in colonic tissues were detected by light microscopy (Nikon, Tokyo, Japan) at × 200.

### Biochemical Analysis

The mouse colon tissue was mixed with PBS in a weight (g)/volume (mL) ratio of 1:9, and thoroughly ground to obtain colon tissue homogenate. The homogenate was then centrifuged at 10,000 × g for 8 min, and the supernatant was collected for further analysis [19]. The levels of TNF-α, IL-1β, and IL-10 in the homogenate supernatant were determined using an ELISA kit manufactured by Jiangsu Meibiao Biotechnology Co. Ltd (Jiangsu, China). The specific operation was conducted by instruction manual, and the optical densities were determined on a Bio-Rad microplate reader (Bio-Rad, USA) at a wavelength of 450 nm. The levels of TNF-α, IL-1β, and IL-10 were calculated according to the standard curve.

### SCFAs Analysis

The cecal contents were placed in a 2-mL centrifuge tube containing 50 μL of 15% (v/v) phosphoric acid, 100 μL of 125 μg/mL internal standard (4-methylpentanoic acid), and 400 μL ether, centrifuged for 1 min, and centrifuged again at 10,000 × g and 4 °C for 10 min. The supernatant was transferred to gas-phase vials and analyzed by gas chromatography-mass spectrometry (GC–MS) (Thermo Fisher Scientific, USA) [20]. For the GC, the samples were injected and then separated using He as the carrier gas (flow rate: 1.0 mL/min, split ratio: 10:1), the inlet temperature was 250 °C. The initial temperature is 90 °C, and the temperature was raised to 120 °C at an increment of 10 °C/min; then, the temperature was raised to 150 °C at an increment of 5 °C/min, raised to 250 °C at an increment of 25 °C/min, and held at 250 °C for 2 min. The MS was scanned in SIM mode at 70 eV [21].

### Western Blotting

The total protein was extracted from the mouse colon tissue according to the method of Wang et al. [22]. The protein concentration was measured by the bicinchoninic acid (BCA) assay, and the same concentration was used in all subsequent experiments. After electrophoresis, the sample was transferred to a membrane made of polyvinylidene fluoride (PVDF). These were then blocked with 3% bovine serum albumin (BSA) at 25 °C for 1 h. The rabbit antibodies used included anti-β-actin (Solarbio, China), anti-TLR4 (No. bs-20595R; Bioss Antibodies Inc., Woburn, MA, USA), anti-MyD88 (No. GTX112987; GeneTex In c., Irvine, CA, USA), anti-PI3K (No. ab40776; Abcam, Cambridge, UK), anti-p-PI3K (No. ab278545; Abcam), anti-Akt (No. N3C2; GeneTex Inc.), anti-Akt (No. GTX128414; GeneTex Inc.), anti-NF-κB (No. ab16502; Abcam), and anti-IκBα (No. ab32518; Abcam). The membrane was incubated at 4 °C overnight, subjected to horseradish peroxidase (HRP)-coupled secondary antibody, and kept in the dark for 1 h. The bands were detected with Cytiva ImageQuant™ LAS 4000 (Cytiva Life Sciences, Marlborough, MA, USA), and the proteins were expressed as a percentage of β-actin.

### Gut Microbial Analysis

Total fecal DNA was extracted with an OMEGA Soil DNA Kit (ONorcross, GA, USA), and 16S rRNA of bacteria was amplified using 338F primer (5′-ACTCCTACGGGAGGCAGCA-3′) and 806R primer (5′-GGACTACHVGGGTWTCTAAT-3′), which were region-specific primers of V3-V4 bacteria. QIIME2 (https://qiime2.org/) and R v. 3.2.0 (Vienna, Austria) were used to analyze the diversity and abundance of the gut microbiota based on relative operational taxonomic units (OTU) at 97% similarity levels. The α-diversity was analyzed using the Chao1, Shannon, and Simpson indices. The β-diversity was evaluated by principal coordinate analysis (PCoA). The relative abundance of the phylum level was used to determine the bacterial community structure, and the difference in genus levels among groups was compared via a heat map.

### Statistical Analysis

The data were presented as means ± standard deviation (SD). They were analyzed with SPSS v. 20.0 (IBM Corp., Armonk, NY, USA), and the graphs were plotted with Origin v. 8.0 (OriginLab, Northampton, MA, USA). Spearman analysis was conducted to investigate the correlation among SCFAs, inflammatory factors, and microorganisms to evaluate the relationship between the gut microbiota and SCFAs and anti-inflammation. Statistically significant differences were evaluated by one-way analysis of variance (ANOVA) followed by the post hoc Bonferroni correction. Significance was defined at the *p* < 0.05 level.

## Results

### *L. plantarum* ELF051 Ameliorated the Pathological Changes in the Colon

**Fig. 1:**
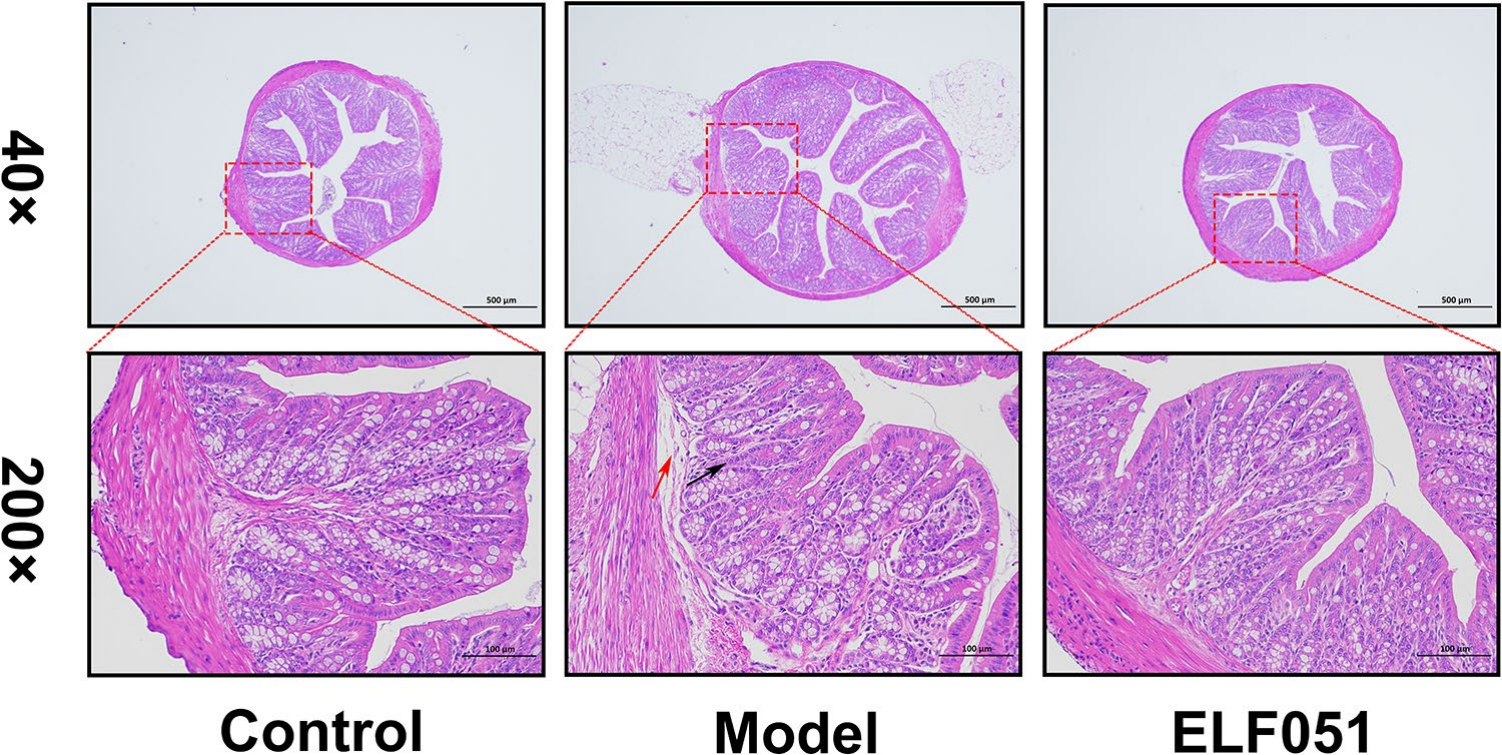
Effect of *L. plantarum* ELF051 on the pathological changes of colon tissue in AAD mice. H&E staining were used to observe the morphological changes in the colon. Black arrow: margin of the intestinal gland was widened; red arrow: connective tissue loose (40 ×, scale bars: 500 µm; 200 ×, scale bars: 100 µm)

H&E staining of the colon tissues revealed distinct structural differences among the experimental groups (Fig. 1). In the control group, the mucosal layer appeared structurally intact with well-preserved mucosal epithelial cells and organized intestinal glands. In contrast, the model group exhibited significant damage, characterized by shedding of mucosal epithelial cells, loosely arranged intestinal glands, and indications of slight edema and inflammatory cell infiltration. However, following the administration of the *L. plantarum* ELF501, the degree of inflammatory cell infiltration decreased and edema was alleviated. Additionally, the loosely arranged intestinal gland structures became tightly arranged. Therefore, *L. plantarum* ELF501 mitigated the intestinal mucosal structural damage caused by antibiotics.

### *L. plantarum* ELF051 Regulates Inflammatory Cytokines in Serum

**Fig. 2:**
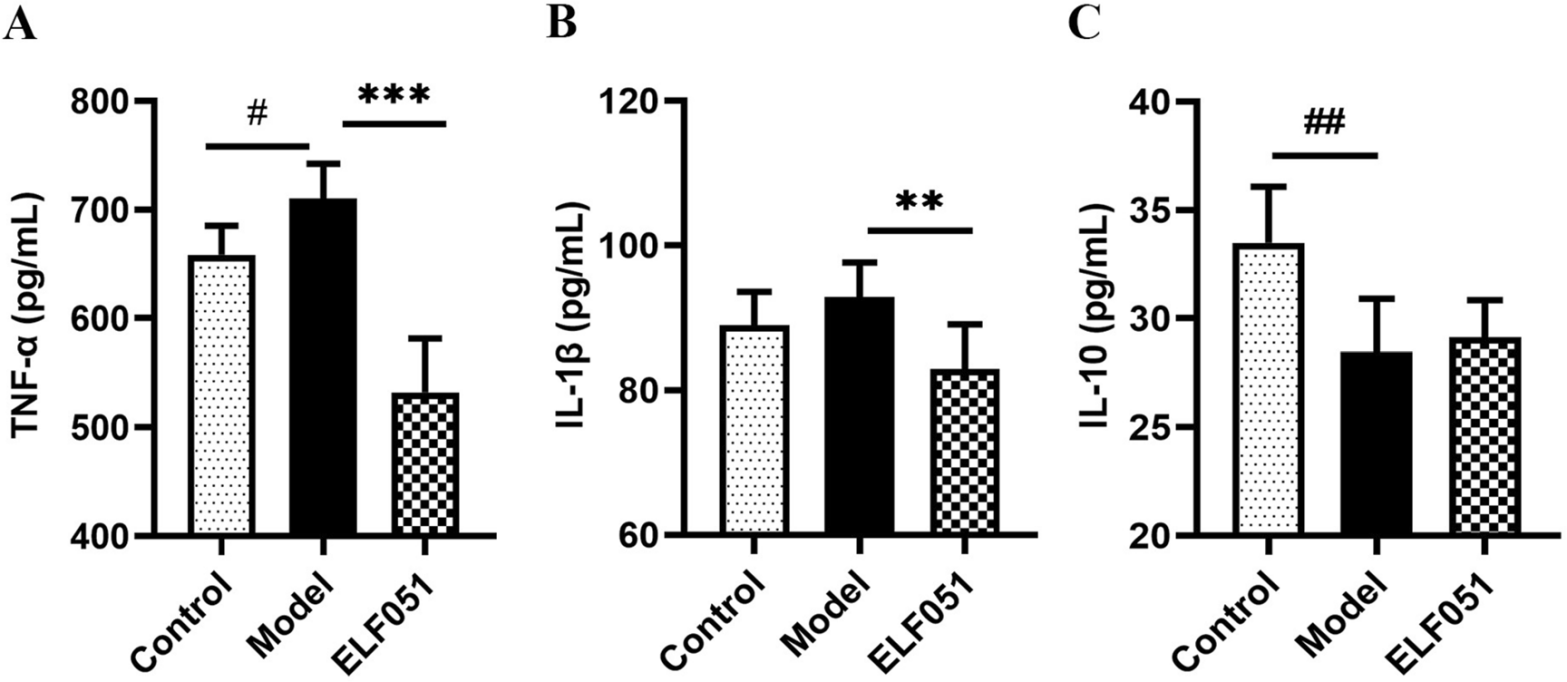
Effect of *L. plantarum* ELF051 on the levels of TNF-α (**A**), IL-1β (**B**), and IL-10 (**C**) in colon tissue of AAD mice. ^#^*p* < 0.05, ^##^*p* < 0.01 vs. control, ^**^p < 0.01, ^***^p < 0.001 vs. model. Data are presented as mean ± S.D, *n* = 6

We measured the TNF-α, IL-1β, and IL-10 levels to determine the impact of *L. plantarum* ELF051 on inflammation (Fig. 2). The TNF-α and IL-1β levels were higher, and the IL-10 level was markedly lower in the model group than in the control group. The TNF-α and IL-1β levels were significantly (*p* < 0.05) lower, while the IL-10 level was higher in the ELF051 group than in the model group.

### *L. plantarum* ELF051 Increased the SCFAs Content

The effect of *L. plantarum* ELF051 on the content of SCFAs in mouse cecal contents was evaluated using GC–MS method. Based on the data presented in Table 1, the model group exhibited significantly lower levels of acetic acid, propionic acid, butyric acid, and total SCFAs showing a significant decrease compared to the control group (*p* < 0.05). After administration of *L. plantar*um ELF051 via gavage in AAD mice, the levels of acetic acid, propionic acid, butyric acid, hexanoic acid, and total SCFAs were increased. Among them, the levels of propionic acid, butyric acid, and total SCFAs showed significant difference when compared to the model group (*p* < 0.05).

**Table 1:** Effect of *L. plantarum* ELF051 on the concentration of SCFAs in cecal contents

	**Acetic acid (μg/g)**	**Propionic acid (μg/g)**	**Butyric acid (μg/g)**	**Isobutyric acid (μg/g)**	**Valeric acid (μg/g)**	**Isovaleric acid (μg/g)**	**Hexanoic acid (μg/g)**	**Total (μg/g)**
Control	1226.28 ± 33.49	466.33 ± 21.25	280.71 ± 23.82	50.44 ± 3.20	62.65 ± 4.19	45.47 ± 5.91	1.05 ± 0.27	2132.72 ± 81.44
Model	952.87 ± 27.40^*^	264.12 ± 25.78^**^	192.76 ± 15.58^*^	49.36 ± 2.58	60.56 ± 2.90	38.65 ± 3.80	0.96 ± 0.02	1559.58 ± 86.81^***^
ELF051	1123.62 ± 35.81^****^	300.43 ± 14.60	274.10 ± 21.05^****^	40.89 ± 3.05	56.62 ± 3.75	38.64 ± 2.01	1.18 ± 0.19	1835.74 ± 134.88^****^

Values are expressed by the mean ± standard deviation (*n* = 6)

^*^p < 0.05; ^**^p < 0.01; ^***^p < 0.001 vs. control; ^****^p < 0.05 vs. model

### *L. plantarum* ELF051 Alleviated Inflammation by Inhibiting the Expression of Key Proteins in the TLR4/MyD88/NF-κB Signaling Pathway

**Fig. 3:**
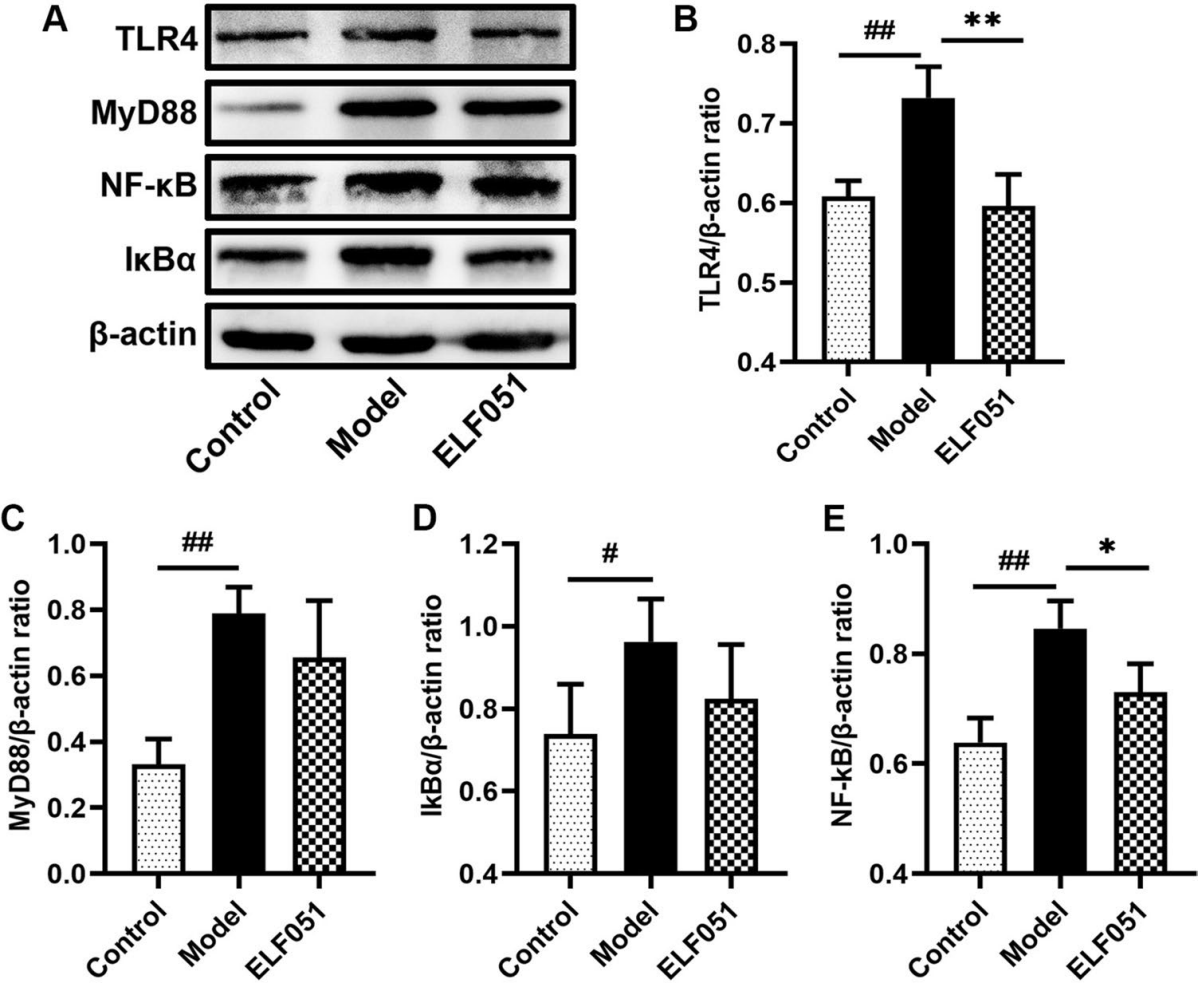
*L. plantarum* ELF051 inhibits the expression of key proteins in the TLR4/MyD88/NF-κB signaling pathway. Protein bands were shown in (**A**), TLR4/β-actin ratio (**B**), MyD88/β-actin ratio (**C**), IκBα/β-actin ratio (**D**), and NF-κB/β-actin ratio (**E**). ^#^*p* < 0.05, ^##^*p* < 0.01 vs. control; ^*^p < 0.05, ^**^p < 0.01 vs. model. Data were presented as the means ± SD, *n* = 6

In order to elucidate the anti-inflammatory effect of *L. plantarum* ELF051 in AAD mice model, we employed Western blot analysis to assess the expression levels of key proteins in the TLR4/MyD88/NF-κB signaling pathway in colonic tissues. Figure 3 shows that TLR4, MyD88, NF-κB, and IκBα were markedly upregulated in the model group compared to the control group but significantly downregulated in the ELF051 group (17.00%, 18.53%, 21.90%, and 14.37%, respectively) compared to the model group. Thus, it can be observed that *L. plantarum* ELF051 can alleviate the inflammatory response in AAD mice by inhibiting the activation of the TLR4/MyD88/NF-κB signaling pathway.

### *L. plantarum* ELF051 Alleviated Inflammation by Inhibiting the Expression of Key Proteins in the PI3Κ/AΚT/NF-κB Signaling Pathway

**Fig. 4:**
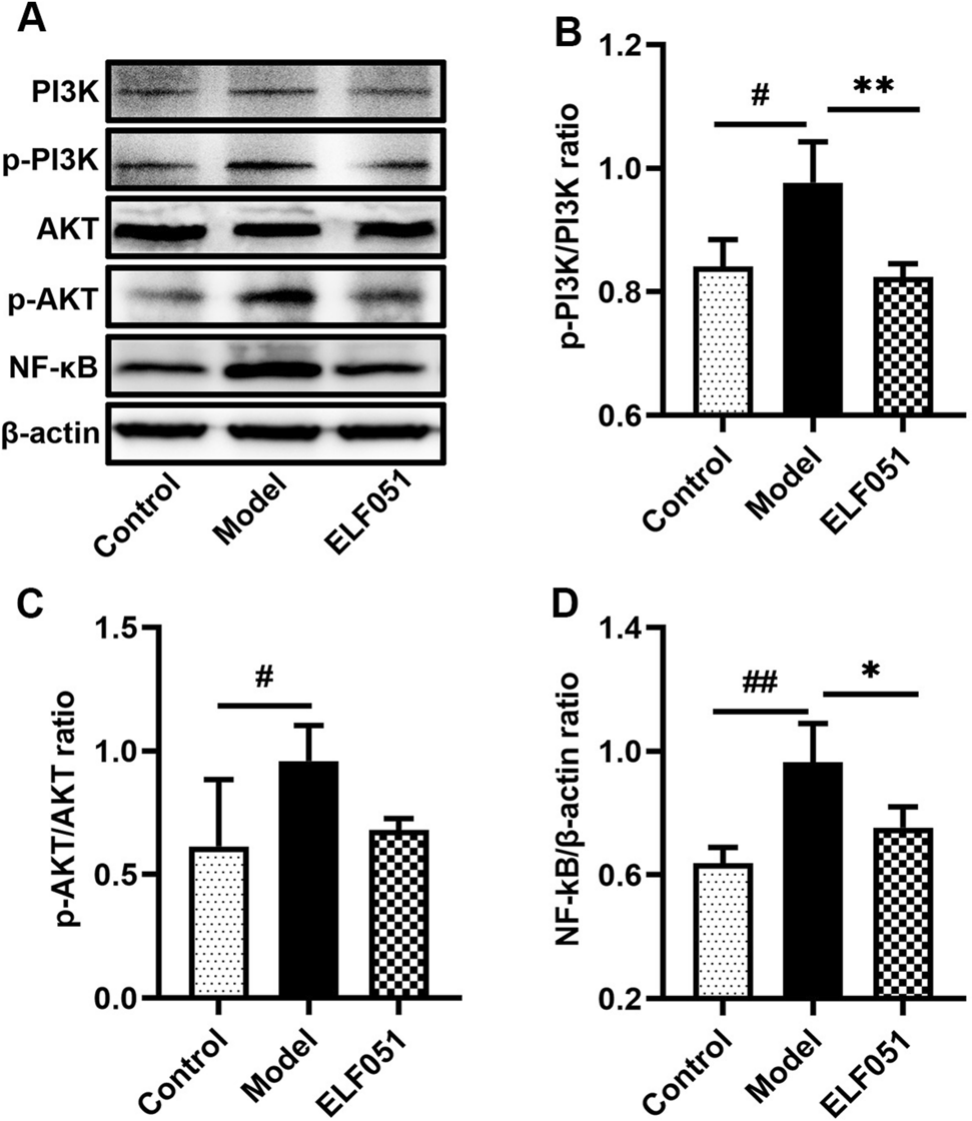
*L. plantarum* ELF051 inhibits the expression of key proteins in the PI3K/AT/NF-κB signaling pathway. Protein bands were shown in (**A**), p-PI3K/PI3Κ ratio (**B**), p-AKT/AΚT ratio (**C**), and NF-κB/β-actin ratio (**D**). Data were analyzed by one-way ANOVA: ^#^*p* < 0.05, ^##^*p* < 0.01 vs. control; ^*^p < 0.05, ^**^p < 0.01 vs. model (*n* = 6 per group). mean ± SD. The β-actin was used as a standard control

We investigated whether the PI3Κ/AΚT/NF-κB pathway is involved in the anti-inflammatory mechanism of *L. plantarum* ELF051 in AAD mice. For this purpose, we compared the expression levels of key proteins in the PI3K/AKT/NF-κB signaling pathway in colon tissue homogenates from different groups. Figure 4 shows that the key proteins of p-PI3Κ, p-AΚT, and NF-κB were significantly upregulated in the model group compared to the control group (*p* < 0.05 or *p* < 0.01). In contrast, the p-PI3Κ/PI3Κ, p-AΚT/AΚT, and NF-κB levels were 29.08%, 15.62%, and 21.90% lower in the ELF051 group than in the model group (*p* < 0.05 or *p* < 0.01). Due to the inhibitory effect of *L. plantarum* ELF051 on the phosphorylation and activation of the PI3K/AKT/NF-κB signaling pathway in colon tissue samples, it is inferred that *L. plantarum* ELF051 exhibits a positive anti-inflammatory effect in AAD mice.

### *L. plantarum* ELF051 Improved Gut Microbiota Dysbiosis in AAD Mice

**Fig. 5:**
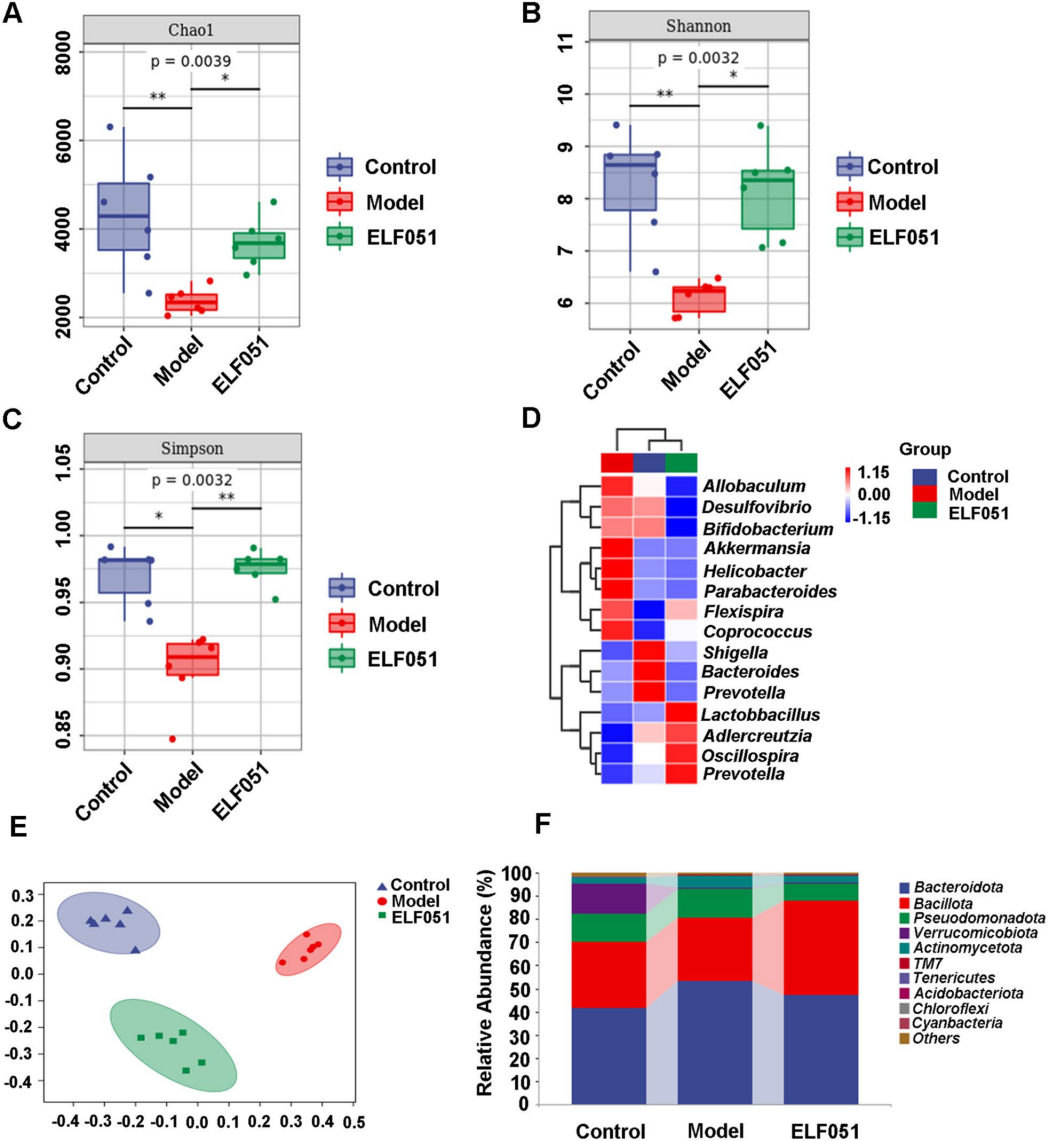
Effects of *L. plantarum* ELF051 on the diversity and composition of the gut microbiota in AAD mice. Chao1, Shannon and Simpson indexes (**A**, **B**, and **C**). Heat map at the genus level (**D**). PCoA analysis based on weighted UniFrac phylogenetic distance matrices (**E**). Species compositions at the phylum level (**F**)

Fecal analysis by 16S rRNA gene sequencing of the V3-V4 region disclosed that *L. plantarum* ELF051 significantly altered gut microbiota richness and diversity compared to the model group. The Chao 1 index indicated that gut microbiota species richness was markedly higher in the ELF051 group than in the model group (Fig. 5A). The Shannon and Simpson indices (Fig. 5B and C) revealed that gut microbiota diversity was higher in the ELF051 group than in the model group. To assess the similarity of gut microbiota in fecal samples, we utilized β-diversity analysis through PCoA. In the PCoA analysis (Fig. 5E), we observed a distinct separation of the model group from the other two groups; the ELF051 group displayed a closer proximity to the control group. The main gut bacterial phyla in all three groups were *Bacillota*, *Bacteroidota*, and *Pseudomonadota* (Fig. 5F). In comparison to the control group, antibiotic treatment resulted in a significant decrease in the relative abundance of *Bacillota*, while increasing the relative abundance of *Bacteroidota* and *Pseudomonadota*. However, following treatment with *L. plantarum* ELF051, the levels of *Bacteroidota* and *Pseudomonadota* were decreased, while the abundance of *Bacillota* showed a significant increase compared to the model group. According to Fig. 5D, treatment with *L. plantarum* ELF051 resulted in a decrease in the abundance of *Allobaculum*, *Desulfovibrio*, and *Akkermansia* compared to the model group. Conversely, the abundance of *Lactobacillus*, *Prevotella*, and *Oscillospira* bacteria showed a significant increase following *L. plantarum* ELF051 treatment. These findings suggest that treatment with *L. plantarum* ELF051 may potentially improve gut microbiota dysbiosis in AAD mice.

### The Correlation Between Microbiota and Inflammatory Factors and Metabolites

**Fig. 6:**
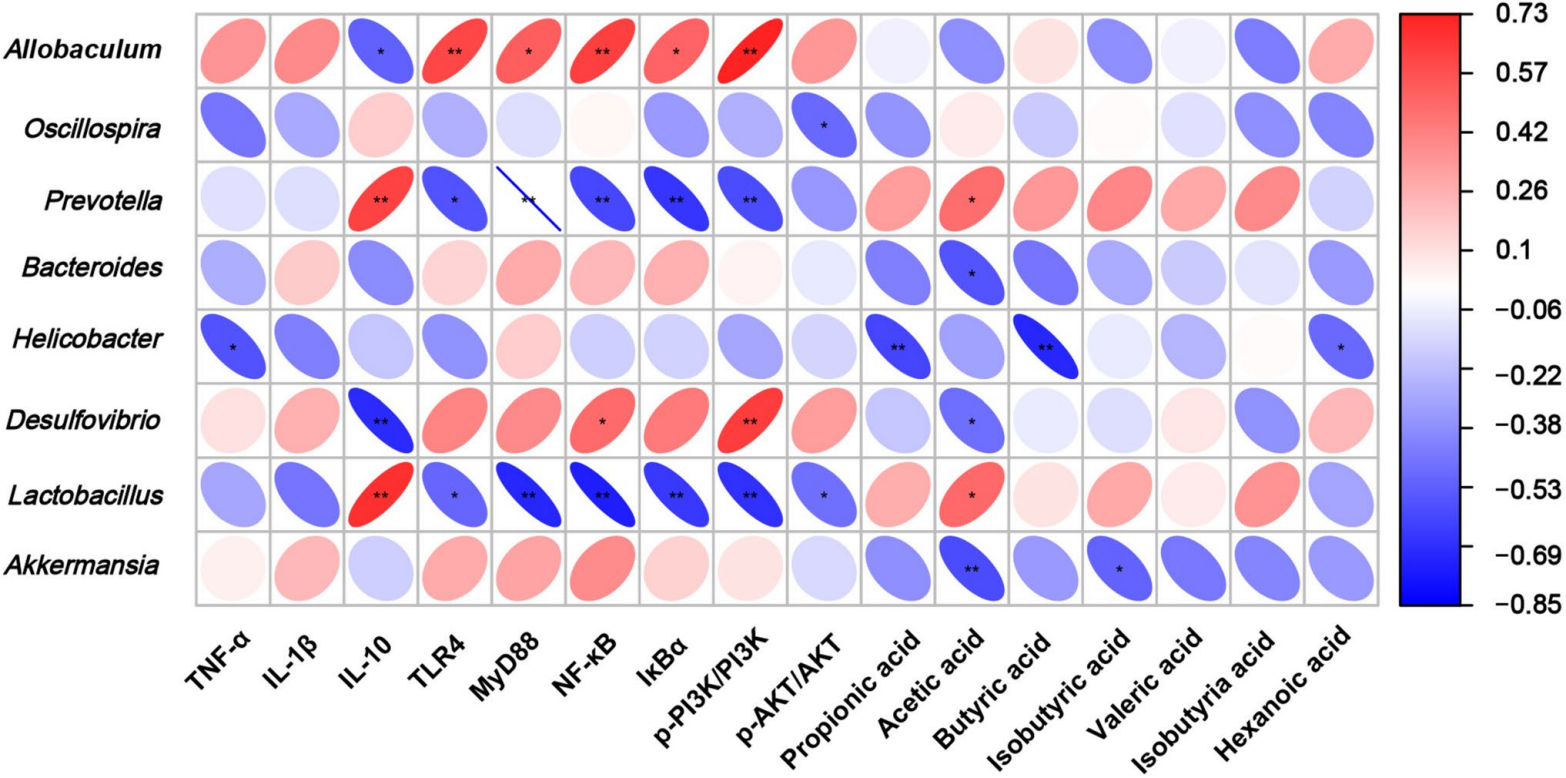
The correlation between gut microbiota and inflammation factors and SCFAs, with red indicating a positive correlation and blue indicating a negative correlation. The symbol “*” denotes the statistical significance of the relationship, ^*^p < 0.05, ^**^p < 0.01 vs model

Spearman’s rank correlation test generated a heatmap that intuitively visualized and disclosed strong associations among microbial species, inflammatory factors, inflammation-related proteins, SCFAs, and others (Fig. 6). *Lactobacillus* abundance was negatively but non-significantly correlated with TNF-α and IL-1β. Conversely, *Lactobacillus* abundance was positively related to IL-10 (*R* = 0.68; *p* = 0.0018). *Lactobacillus* abundance was negatively related to TLR4 (*R* = − 0.509; *p* = 0.031), MyD88 (*R* = − 0.719; *p* = 0.00077), NF-κB (*R* = − 0.742; *p* = 0.0004), IκBα (*R* = − 0.664; *p* = 0.0027), p-PI3K/PI3K (*R* = − 0.682; *p* = 0.0018), and p-Akt/Akt (*R* = − 0.474; *p* = 0.047). *Lactobacillus* was also positively related to the acetic acid content (*R* = 0.496; *p* = 0.036). SCFAs detected were closely associated with *Lactobacillus*, *Oscillospira*, and *Prevotella*. It can be inferred from the foregoing data that *L. plantarum* ELF051 may downregulate the pro-inflammatory factors, increase the inflammation-related protein content, and elevate the SCFAs concentrations by regulating the composition of the intestinal microbiota in AAD mice.

## Discussion

AAD has a complex pathogenesis. Antibiotic misuse or overuse can alter the gut microbiota and SCFAs profile, induce inflammation, and cause AAD [23]. In the present study, a mouse AAD model was constructed through intragastric amoxicillin, clindamycin, and streptomycin administration. This treatment altered the intestinal structure and microbiota and changed the gut SCFAs content. It also upregulated pro-inflammatory and downregulated anti-inflammatory cytokines and lowered the inflammation-related protein content in the intestine. Intragastric administration of the probiotic *L. plantarum* ELF051 improved AAD symptoms. Therefore, ELF051 could be efficacious in AAD therapy.

SCFAs are important end products of gut microbiota metabolism, and they are generated through the fermentation of indigestible carbohydrates. SCFAs may have potential anti-inflammatory and reparative effects on intestinal damage [24]. The present study showed that administration of *L. plantarum* ELF051 increased the levels of SCFAs in the intestine. Liu et al. found that *Bacillus coagulans* upregulated the levels of SCFAs in mice with colitis, and the increase in SCFAs played an anti-inflammatory role by regulating intestinal immune activity, and enhancing the function of the epithelial barrier [25]. Therefore, we infer that the restoration of intestinal pathological damage and a further alleviation of AAD-related symptoms by *L. plantarum* ELF051 are related to the increase of SCFAs levels. In addition, we observed a positive correlation between the amount of *Bacteroidota* and *Bacillota* and the concentrations of propionic and butyric acid in the gut (Fig. 5F). It is reported that butyric acid is produced by *Bacillota* and acetic acid is produced by *Bacteroidota* [26]. Research indicates that butyric acid is predominantly produced by *Bacillota* in the intestine and plays a vital role in maintaining intestinal health. Butyric acid has been shown to enhance intestinal permeability and promote intestinal health, contributing to the alleviation of diarrhea [27]. Treatment with *L. plantarum* has been shown to increase the levels of butyric acid. Shi et al. showed that treatment with *L. casei* CGMCC 12435 increased butyric acid concentration [28]. Therefore, *L. plantarum* ELF051 has the potential to improve AAD by enhancing the production of SCFAs.

Inflammation is a natural defense mechanism against harmful stimuli. Pro-inflammatory cytokines in general, and TNF-α in particular, are produced in abundance during this process. TNF-α activates the NF-κB pathway and upregulates other pro-inflammatory cytokines, thereby exacerbating the inflammatory response [29]. The present work demonstrated that the probiotic *L. plantarum* ELF051 significantly downregulated TNF-α and IL-1β while upregulating IL-10 in the mouse colons. These findings were consistent with those reported in previous studies. *L. plantarum* HNU082 and *B. adolescentis* also attenuated the inflammatory response in a DSS-induced colitis by downregulating TNF-α and IL-1β while upregulating IL-10 [30, 31]. Hence, the ability of *L. plantarum* ELF051 to decrease pro-inflammatory and increase anti-inflammatory factors may explain its therapeutic efficacy against AAD. A growing body of evidence indicates that the TLR4/MyD88/NF-κB pathway is implicated in the amelioration of diarrhea. The TLR4 signaling pathway is subdivided into the MyD88-dependent and MyD88-independent pathways [32]. Tong et al. showed that *L. rhamnosus* GG play a key role in the DSS-induced inflammatory response through its downregulation of the TLR4-MyD88 axis [33]. The MyD88-independent signaling pathway generates abundant interferon (IFN)-γ which is also associated with colonic inflammatory activity. Thus, we analyzed the MyD88-dependent pathway to elucidate the mechanism by which *L. plantarum* ELF051 ameliorates AAD. *L. plantarum* ELF051 significantly downregulated TLR4, MyD88, NF-κB, and IκBα. An earlier experiment demonstrated that *L. rhamnosus GG* Effector Protein HM0539 substantially mitigated inflammation and downregulated IL-6, IL-1β, and TNF-α in murine colitis. IL-6 and TNF-α are important mediators of the TLR4/MyD88/NF-κB pathway [34]. Another study showed that *L. plantarum* AR113 alleviated dextran sulfate sodium (DSS)–induced colitis in mice by downregulating the TLR4/MyD88/NF-κB pathway in the colon [35]. The results of this study indicated that in AAD mice, there was an increase in the expression of PI3K and AKT proteins, activating NF-κB, leading to an increase in the levels of phosphorylated IκBα and p65, thereby promoting the secretion of cytokines IL-1β, IL-6, and TNF-α. These cytokines have pro-inflammatory effects and contribute to the development of diarrhea by causing intestinal inflammation [36, 37]. However, after treatment with *L. plantarum* ELF051, NF-κB activation was inhibited, and regulated the PI3K/AKT/NF-κB signaling pathway, leading to a reduction in the secretion of inflammatory cytokines. Consist with previous study, gavage with the probiotic powder VSL#3 also exerted anti-inflammatory effects by inhibiting the PI3K/AKT and NF-κB pathways in the DSS induced rat colitis model [38].

Antibiotic use may disrupt the gut microbiota, leading to an increase in the abundance of harmful bacterial pathogens such as *Actinomycetota* and *Pseudomonadota*. Genera within these phyla are known to cause various types of infections in humans, animals, and plants, and in severe cases, affect host immunity [39–41]. In this study, the administration of *L. plantarum* ELF051 resulted in notable alterations in the abundance and diversity of the intestinal microbiota in mice. Specifically, the abundance of *Pseudomonadota*, *Bacteroidota*, and *Actinomycetota* decreased, while the abundance of *Bacillota* exhibited a reversal. These findings align with previous published reports [17]. At the genus level, the administration of *L. plantarum* ELF051 resulted in an increase in the abundance of *Lactobacillus*, *Oscillospira*, and *Prevotella*, while decreasing the relative abundance of *Allobaculum*, *Desulfovibrio*, and *Akkermansia*, compared to the model group. Guo et al. reported that antibiotic-associated diarrhea was induced by ceftriaxone in mice, the relative abundance of *Lactobacillus* and *Prevotella* was increase after gavage with apple juice fermented by *L. plantarum* CICC21809, which is consistent with the results of this study [42]. A Pearson’s correlation analysis disclosed the associations among microbiota and inflammatory mediators and SCFAs (Fig. 6), and the results indicated a close correlation between *Lactobacillus* and inflammatory mediators and SCFAs. Our findings suggest that increasing abundances of *Lactobacillus* spp. and the content of lactic acid and the other SCFAs they produced help establish and maintain homeostasis in the gut microbiota environment and attenuate the intestinal inflammatory response.

## Conclusion

In conclusion, the current research findings suggest that the probiotic *L. plantarum* ELF051 has positive effects on AAD. *L. plantarum* ELF051 by improving the tissue morphology, normalizing the levels of inflammatory cytokines, increasing the production of SCFAs in the colon, and modulating the gut microbiota. In addition, *L. plantarum* ELF051 regulates the TLR4/MyD88/NF-κB and PI3K/AKT/NF-κB signaling pathways, thereby alleviating inflammation. These findings may provide a novel therapeutic approach for the prevention and treatment of AAD.

## Data Availability

All 16 s rRNA Illumina amplicon sequencing data provided in this study can be publicly obtained in the Sequence Read Archive (SRA) of The National Center for Biotechnology Information (NCBI) under the accession number SRP PRJNA973244.
